# Plants of the Cerrado with antimicrobial effects against *Staphylococcus* spp*.* and *Escherichia coli* from cattle

**DOI:** 10.1186/s12917-018-1351-1

**Published:** 2018-01-30

**Authors:** Izabella Carolina de O. Ribeiro, Emanuelly Gomes A. Mariano, Roberta T. Careli, Franciellen Morais-Costa, Felipe M. de Sant’Anna, Maximiliano S. Pinto, Marcelo R. de Souza, Eduardo R. Duarte

**Affiliations:** 1 Instituto de Ciências Agrárias, Universidade Federal de Minas Gerais, Avenida Universitária, 1000, Bairro Universitário, Montes Claros, Minas Gerais CEP 39401-790 Brazil; 2 Escola de Medicina Veterinária, Universidade Federal de Minas Gerais, Av. Antonio Carlos, 6627 Pampulha, Belo Horizonte, MG CEP 31270-901 Brazil; 3 Instituto de Ciências Agrárias, Universidade Federal de Minas Gerais, Av Universitária 1000, Bairro Universitario, Montes Claros, MG 39400-006 Brazil

**Keywords:** Antibacterial, Brazilian savannah, Colibacillosis, Mastitis, Medicinal plants, *Staphylococcus Aureus*, *Staphylococcus haemolyticus*

## Abstract

**Background:**

Both diarrhea in calves and mastitis in cows limit cattle production. The bacteria involved in these diseases have shown multi-resistance to antimicrobials, however plant metabolites therefore can provide an alternative method of control. This study selected and characterized Cerrado plant extracts showing inhibitory effects against *Escherichia coli* and *Staphylococcus* spp. from cattle. Thirteen leaf extracts were initially screened and diameters of inhibition zones produced against the pathogens were recorded using an agar disk diffusion method. Total condensed tannin contents were determined and antibacterial activities were analyzed after tannin removal from the five selected extracts. The minimum inhibitory concentrations (MIC) and minimum bactericidal concentrations (MBC) were evaluated by macro-dilution antimicrobial susceptibility tests, and the extracts were characterized by high performance liquid chromatography.

**Results:**

Inter- and intra-specific bacterial variations in the susceptibility to the extracts were detected. The aqueous extract (AE) from *Caryocar brasiliense* Cambess. leaves produced larger inhibition zones against *E. coli* strains than did other selected extracts. However, the AE from *Schinopsis brasiliensis* was the most effective against *Staphylococcus* spp. strains (*P* < 0.001). The MIC of ethanolic extracts (EE) from *C. brasiliense* (0.27 mg/mL) and *S. brasiliensis* (0.17 mg/mL) were lower than those of other extracts. The MIC and MBC of the *Annona crassiflora* EE were 6.24 mg/mL for all bacteria. Flavonoids were the main metabolites detected in the *A. crassiflora* EE as well as in the AE and EE from *C. brasiliense,* while tannins were the main metabolites in the *S. brasiliensis* leaf extracts.

**Conclusion:**

The AE from *C. brasiliense* was more effective against Gram-negative bacteria, while the AE from *S. brasiliensis* was more effective against Gram-positive bacteria. *A. crassiflora* EE and *S. brasiliensis* extracts are potent bactericide. After removal of the tannins, no antimicrobial effects were observed, indicating that these metabolites are the main active antibacterial components.

## Background

Diseases must be prevented or controlled in order to achieve a sustainable and viable production of ruminants. Diarrhea is the most common pathology in young calves and *Escherichia coli* represents one of its main etiological agents [1, 2]. Mastitis, caused by the *Staphylococcus* spp., is the most important disorder in cows, and leads to reduced milk production and increased production costs [3, 4]. These bacteria have shown multi-resistance to antimicrobials in different continents and present a public health risk [2, 5–7].

Plant metabolites are considered alternative control agents for the reduction of resistant microorganisms and antimicrobial residues in foods of animal origin [5–7]. Scientific literature has frequently reported the inhibitory action of certain plant extracts against bacteria in humans [7, 8]. However, few studies have showed effective plant extracts inhibiting microorganisms from ruminants or other animals. Extracts from *Solanum paniculatum* L. (Jurubeba) and *Punica granatum* L. (Romã) display antibacterial effects against microorganisms that cause bovine mastitis [9], and *Rhodomyrtus tomentosa* L. (rose myrtle) leaf extract shows potent antibacterial activity against *Staphylococcus aureus* in milk [10]. Tannins are the main antimicrobial metabolites in vegetal extracts, and also inhibit enzymes and alter metabolism via membrane or cell wall interactions [11].

The Cerrado, a type of savannah present in South America, is native to more than 10,000 plant species that contain natural products for phytotherapy [12]. With regards to alternative antibacterial agents, four medicinal plants from the Brazilian Cerrado have been shown to inhibit the growth of *S. aureus* [13], and the leaf extract from *Schinopsis brasiliensis* Engl. is effective against multidrug-resistant *S. aureus* [14].

However, the antimicrobial activity of plant species from the Brazilian Cerrado against animal pathogens has not been fully explored. Extracts from these plants that show antibacterial effects could favor the alternative controls, thereby reducing pathogen multi-resistance. In organic animal production systems, the use of these extracts would enable the production of foods free from antimicrobial residues, thus increasing the value of these animal products.

In this study, plant species commonly found in the Cerrado were selected and their antimicrobial activities were evaluated against isolates of *E. coli* and *Staphylococcus* spp. from cattle. To identify the main antibacterial components, these extracts were characterized by high performance liquid chromatography (HPLC), and inhibitory activity was analyzed after tannin removal.

## Methods

### Microorganisms

The antibacterial effects of the plant extracts were evaluated against three *Staphylococcus* isolates (S178, S135, and S182) from cows with mastitis. These bacteria were isolated and cultured on mannitol salt agar, and evaluated based on colony characteristics, Gram staining, catalase reaction, and coagulase test. We also assessed the inhibitory effects of the extracts on two *E. coli* isolates (E2 and E3) from the feces of dairy calves with diarrhea. These were isolated and cultured on MacConkey agar and colony characteristics, Gram staining, and catalase reactions were evaluated. These animals were raised on an experimental farm in northern Minas Gerais, Brazil. In addition, the human clinical isolates *S. aureus* ATCC 25923 and *E. coli* ATCC 25922 were included as reference strains. All bacteria were cultured in Brain Heart Infusion (BHI) broth, and subsamples were stored at − 80 °C after glycerol inclusion (1:1).

DNA from these bovine isolates was extracted and amplified as described by Chapaval et al. [15]. DNA samples were amplified via polymerase chain reaction (PCR) using primers 27F (5′-AGAGTTTGATCCTGGCTCAG-3′) and 1492R (5′-GGTTACCTTGTTACGACTT-3′), as described by Lane [16]. 16S ribosomal RNA (rRNA) was sequenced following the method described by Sanger [17], using the automatic sequencer MegaBACE® 1000 (GE Life Sciences, USA), according to Reysenbach et al. [18]. 16S rRNA gene sequencing was verified using the SeqScanner Software® v1.0 (Applied Biosystems, USA), and the results were compared online by BLAST (database from NCBI - https://blast.ncbi.nlm.nih.gov/Blast.cgi). The bacteria species were identified with a similarity level of at least 99%.

### Antibacterial susceptibility

The procedure for the agar disk diffusion method was performed in triplicate, according to recommendations by the National Committee for Clinical Laboratory Standards (NCCLS) [19]. For *Staphylococcus* strains, the following antimicrobial discs were added onto the medium surface: chloramphenicol, 30 μg; erythromycin, 15 μg; vancomycin, 30 μg; oxacillin, 1 μg; gentamicin, 10 μg; tetracycline, 30 μg; clindamycin, 2 μg; and penicillin, 10 μg. For *E. coli* strains, the following were used: chloramphenicol, 30 μg; ampicillin, 10 μg; gentamicin, 10 μg; ciprofloxacin, 5 μg; tetracycline, 30 μg; and norfloxacin, 10 μg, as described by the NCCLS [19]. For quality control purposes, the strains ATCC 25923 and ATCC 25922 were used. All plates were incubated at 35 °C for 24 h, and inhibition zones (mm) were measured and the bacteria were classified as resistant or sensitive according to the NCCLS guidelines [19].

### Plant extracts

Plant leaves were collected from April to June at the Institute of Agricultural Sciences, UFMG, in Montes Claros, Minas Gerais, Brazil. This region is located at latitude 16°51′ and longitude 44°55′, and the climate is tropical and humid with dry summers (A) according to Köppen classification [20].

Vegetal materials were collected from *Caryocar brasiliense* Camb. (Caryocaraceae), *Annona crassiflora* Mart. (Annonaceae), *S. brasiliensis* Engl. (Anacardiaceae), *Piptadenia viridiflora* (Kunth) Benth. (Fabaceae), *Serjania lethalis* A.St.-Hil. (Sapindaceae), *Casearia sylvestris* (Flacourtiaceae), and *Ximenia americana*L. (Olacaceae). Plant samples were deposited in the Montes Claros Herbarium of Universidade Estadual de Montes Claros, as voucher specimens 338, 1492, 377, 2283, 2249, 3008, and 211, respectively.

The leaves were carefully inspected, and those with gross lesions or damage were discarded. Selected leaves were dehydrated under forced air circulation (TE 394/4, Tecnal Equipamentos Científicos Tecnal, Piracicaba, SP, Brazil) at 38 °C for 72 h, crushed in a blender, and stored inside paper bags in the dark at − 4 °C [11, 21].

Aqueous extracts (AEs) were produced by placing the ground dried leaves in a distilled water bath at 40 °C for 60 min. Ethanolic extracts (EEs) were obtained from macerated dried leaves held in absolute ethanol in amber-colored glass containers in the dark for seven days. Extracts were filtered through a gauze funnel and subsequently evaporated at 40 °C for 48 h under forced air circulation until completely dry and stored at 4 °C until use [11]. In this study, both EEs and AEs were completely soluble in distilled water and did not require any other solvents for antimicrobial analysis.

Subsamples of extracts were subjected to tannin extraction according to the method described by Nyman et al. [22]. Extracts were dissolved in water (1 g per 20 mL) at 90 °C and cooled to room temperature. After reaching a temperature of 30 °C, 0.2 μL 10% NaCl was added, and 1 mL of this solution was combined with 4 mL 1% gelatin solution before centrifuging at 1800×g for 6 min. Supernatants were used to assess the effects of tannin-free extracts.

### Characterization of extracts

A Waters Alliance 2695 HPLC system comprising a quaternary pump, auto-sampler, photodiode array detector (DAD) 2996, and Waters Empower Pro data handling system (Waters Corporation, Milford, Connecticut, USA) were used for extract characterization. Analyses were performed on a LiChrospher 100 RP-18 column (250 × 4 mm, 5 mm; Merck, Darmstadt, Germany) combined with a LiChrospher 100 RP-18 guard column (4 × 4 mm, 5 mm; Merck) at 40 °C. Water (A) and acetonitrile (B) were used as eluents, both containing 0.1% (*v*/v) H_3_PO_4_ at a flow rate of 1.0 mL/min as follows: 0 min, 95% A and 5% B; 60 min, 5% A, 95% B, followed by 10 min isocratic elution. Solvents used were of HPLC grade (Merck, Germany) and were degassed by sonication before use. Chromatograms were obtained at 210 nm, and the UV spectra were recorded online from 190 to 400 nm.

The dried crude extracts were dissolved in methanol (HPLC-grade), ultrapure water, or hydroethanolic solutions according to their solubility, to concentrations of 10 mg/mL. After centrifugation at 8400×g for 10 min, 10 mL sample were automatically injected into the apparatus.

The total condensed tannin (proanthocyanidins) content of the extracts was determined by measuring the absorbance of cyanidin chloride resulting from acid-catalyzed solvolysis with n-BuOH/HCl 12 M (95:5) at 540 nm, according to the method described by Hiermann et al. [23]. Each sample was analyzed in triplicate and the total condensed tannin content, expressed as cyanidin chloride, was calculated using the following formula:

Condensed tannins % = Absorbance (sample) − Absorbance (blank) × 4.155/sample weight (g).

### Selection of plant extracts with inhibitory effects

Seven ethanolic and six aqueous extracts were diluted with distilled water at 0.1 g extract/mL and vortexed for 3 min. Extracts were used immediately after this preparation. Antibacterial activity was determined using the agar disk diffusion method [19, 24].

A loopful of bacteria was inoculated onto BHI agar under sterile conditions, and incubated at 37 °C for 24 h. Turbidity equivalent to a 0.5 McFarland standard was used as a reference to adjust for approximately 10^8^ colony-forming units (CFU)/mL. One hundred microliters of freshly prepared inoculum suspension were spread on Mueller-Hinton agar using sterile swabs [19]. Eight microliters of extract solution was added to 6-mm paper filter disks, allocated onto the surface of the seeded plates, and incubated at 35 °C for 24 h. Inhibition zones were then measured using a digital caliper [8]. In this screening assay, all procedures were performed in duplicate.

Based on the largest zones of inhibition and the broadest spectrum of action, five extracts were selected, both with and without tannins. These extracts were filtered through a 0.2-μm Millipore membrane and aliquots were then submitted to dry matter (dm) determination in an oven at 105 °C, in order to standardize them at 1.58 mg dm/mL. Paper filter disks with sterile saline solution (without extract) and discs containing the extracts incubated without bacteria were used as controls [8, 24]. The experiment was designed in a factorial arrangement (5 extracts × 7 bacterial strains) and all procedures were performed in triplicate. The inhibition zone averages were compared by the analysis of variance using Scott-Knott’s test at the 5% significance level, using the System for Statistical Analysis software (SAEG 9.1).

### Minimum inhibitory concentration (MIC) and minimum bactericidal concentration (MBC)

After filtration of the extracts, we determined the MIC necessary to inhibit the growth of the microorganism by macro-dilution in Mueller-Hinton broth, as described by the NCCLS [25].

Using a 1:2 dilution with an equal volume of medium, the final concentrations (6.24–0.01 mg/mL) were evaluated. However, for MBC determinations of the *C. brasiliensis* extracts were also evaluated concentrations up to 40 mg/mL. Extract solutions were therefore prepared at double the final concentration [25]. Completing at final volume of 5 mL, 120 μL of bacteria inoculum prepared as reported above were added together with 2.48 mL Mueller Hinto broth and 2.5 mL of extract solution.

For the controls, we used growth control tubes containing broth without extract for each bacterium tested, tubes without bacteria containing broth alone or added of extracts. All tubes were incubated at 35 °C for 24 h in a thermo-shaker incubator (Novatécnica, São Paulo, SP, Brazil) to ensure homogenization. After this period, bacterial growth was assessed using 125 μL 0.5% triphenyl tetrazolium chloride (TTC) solution, which indicates cellular multiplication through the development of a reddish color in the presence of viable cells, thus enabling MIC determination [26].

Subsequently, the MBC was determined, which represents the lowest concentration of the extract necessary to achieve complete suppression of bacterial growth. One hundred-microliter aliquots from the tubes used for the MIC assay and the control tubes without extracts were inoculated on Mueller-Hinton agar and incubated at 37 °C for 24 h. The absence of bacterial growth on the agar plate was evaluated to determine MBC, and the experiment was carried out in triplicate.

## Results

### Characterization of bacterial isolates

Using molecular analysis, we successfully identified isolate S178 as *S. aureus* and isolates S135 and S182 as *S. haemolyticus* (99.9% similarity). Strains E2 and E3 were identified as *E. coli,* as shown in Table 1.

**Table 1:** Bacterial identification by 16S rDNA sequencing and classification according to BLAST (NCBI database)

Isolates	Origen	Number of analyzed nucleotides	Identification with similarity > 99%
S178	Cow whit matitis	551	*Staphylococcus aureus subsp. aureus* strain *NCTC 8325*
S135	Cow whit matitis	553	*Staphylococcus haemolyticus* strain *JCSC1435*
S182	Cow whit matitis	550	*Staphylococcus haemolyticus* strain v *JCSC1435*
E2	Calf with diarrhea	590	*Escherichia coli str. K-12 substr.* strain *MG1655*
E3	Calf with diarrhea	574	*Escherichia coli str. K-12 substr.* strain *MG1655*

Isolate S178 (*S. aureus*) was resistant to erythromycin, clindamycin, oxacillin, penicillin, tetracycline, and vancomycin. However, *S. haemolyticus* isolates were sensitive to all antimicrobials tested in gram-positive strains. All *E. coli* strains were resistant to tetracycline, while isolate E3 was also resistant to ampicillin and gentamicin (Table 2).

**Table 2:** Antimicrobial sensitivity profiles for *Staphylococcus aureus*, *Staphylococcus haemolyticus,* and *Escherichia coli* isolates from cattle and standard strains

	*S. haemolyticus*		*S. aureus*		*E. coli*		
Antibacterial	S135	S182	S178	ATCC	E2	E3	ATCC
Chloramphenicol	S	S	S	S	S	S	S
Erythromycin	S	S	R	S	–	–	–
Ampicillin	–	–	–	–	S	I	S
Vancomycin	S	S	R	R	–	–	–
Oxacillin	S	S	R	R	–	–	–
Gentamicin	S	S	S	S	S	I	I
Ciprofloxacin	–	–	–	–	S	S	S
Tetracycline	S	S	R	S	R	I	R
Clindamycin	S	S	R	R	–	–	–
Penicillin	S	S	R	R	–	–	–
Norfloxacin	–	–	–	–	S	S	S

chloramphenicol 30 μg, erythromycin 15 μg, ampicillin 10 μg, vancomycin 30 μg, oxacillin 1 μg, gentamicin 10 μg, ciprofloxacin 5 μg, tetracycline 30 μg, clindamycin 2 μg, penicillin 10 μg e norfloxacin 10 μg. *S* Sensitive, *I* Intermediate, *R R*esistant, according to NCCLS (2005)

### Selection of antimicrobial extracts from Cerrado plants

The initial screen revealed that all extracts presented inhibitory effects on at least one of the evaluated bacterial strains. However, the EE from *X. americana* did not show any inhibitory effects on *E. coli* or *S. haemolyticus* strains (Table 3). The EE and AE from *C. brasiliense* and EEs from *A. crassiflora*, *S. brasiliensis,* and *S. lethalis* presented antagonism against all *Staphylococcus* spp. strains. The different phenological stages of *C. brasiliense* produced leaf extracts showing inhibitory effects against *S. aureus* and *S. haemolyticus* (Table 3). Inhibition zone measurements were not associated with tannin content in the extracts tested (*P* > 0.05, Pearson correlation).

**Table 3:** Selection of vegetal extracts according to inhibition zones (mm) produced in *Staphylococcus aureus, Escherichia coli,* and *Staphylococcus haemolyticus* after addition of extracts from Cerrado plant leaves in an agar diffusion test

Vegetal species	Extracts	Tannin content (%)	*S. haemolyticus*		*S. aureus*		*Escherichia coli*		
			135 AE	182	178	ATCC	E2	E3	ATCC
*Caryocar brasiliense* in flowering	Ethanolic	1.99 ± 0.12	26.7 ± 2.5	14.1 ± 1.9	30.2 ± 3.4	20.2 ± 3.3	0.0	0.0	19.0 ± 1.9
*C.brasiliense* in flowering	Aqueous	1.37 ± 0.08	23.5 ± 3.5	14.3 ± 1.8	21.8 ± 2.4	19.3 ± 2.3	0.0	0.0	14.2 ± 2.7
*C. brasiliense* in fruiting	Aqueous	1.25 ± 0.02	24.3 ± 2.6	14.0 ± 2,7	21.0 ± 2.0	23.8 ± 2.0	0.0	0.0	16.4 ± 3.7
*C. brasiliense* flowerless and fruitless	Aqueous	2.66 ± 0.22	20.2 ± 2.0	11.1 ± 2.6	16.2 ± 4.1	15.8 ± 3.1	0.0	0.0	13.8 ± 5.0
*Annona crassiflora*	Aqueous	2.59 ± 1.47	12.8 ± 2.3	0.0	15.9 ± 2.7	13.1 ± 4.0	0.0	8.0 ± 3.0	0.0
*Annona crassiflora*	Ethanolic	4.20 ± 2.38	9.3 ± 2.1	14.5 ± 1.2	15.9 ± 3.1	14.6 ± 3.4	0.0	19.8 ± 4.1	11.1 ± 3.4
*Piptadenia viridiflora*	Aqueous	0.23 ± 0.01	16.4 ± 2.3	0.0	19.1 ± 3.1	0.0	0.0	11.5 ± 2.7	29.9 ± 5.5
*Piptadenia viridiflora*	Ethanolic	1.75 ± 0.21	9.0 ± 0.95	0.0	13.3 ± 3.3	17.1 ± 3.1	0.0	0.0	27.0 ± 4.9
*Schinopsis brasiliensis*	Aqueous	0.16 ± 0.37	16.4 ± 2.3	18.0 ± 0.3	24.1 ± 2.9	20.8 ± 3.1	9.4 ± 1.0	0.0	22.3 ± 3.3
*Schinopsis brasiliensis*	Ethanolic	0.72 ± 0.34	21.7 ± 3.6	21.2 ± 4.3	22.5 ± 2.7	22.8 ± 2.3	0.0	16.2 ± 3.4	20.6 ± 5.6
*Serjania lethalis*	Ethanolic	6.37 ± 0.29	13.2 ± 2.5	11.0 ± 2.2	14.5 ± 3.0	18.5 ± 4.7	0.0	10.0 ± 3.0	11.7 ± 4.6
*Casearia sylvestris*	Ethanolic	7.36 ± 0.54	8.2 ± 1.3	0.0	10.9 ± 2.0	7.5 ± 4.3	0.0	0.0	15.0 ± 3.8
*Ximenia americana*	Ethanolic	0.29 ± 0.02	0.0	0.0	0.0	24.2 ± 4.5	0.0	0.0	0.0

The EE from *A. crassiflora*, and EEs and AEs from *S. brasiliensis* and *C. brasiliense* were selected owing to their inhibitory effects, which produced large inhibition zones. We also considered whether plants acted on one bacterial species or on both, the latter showing a broader spectrum of action. These extracts were sterilized by filtration and concentrations were standardized to 1.58 mg/mL to compare their effects via diffusion tests in agar.

When evaluating the effects of the five selected extracts on the seven bacterial strains, differences between the type of extract, the plant species, and the strain and species of bacteria were detected (*P* < 0.001). Consequently, interactions between the type of extract evaluated and the bacterial strain were also significant (*P* < 0.01, Table 4). After removal of the tannins, no inhibitory effects were observed for the selected extracts (Table 4).

**Table 4:** Average inhibition zones (mm) produced in an agar diffusion test in *Staphylococcus aureus*, *Staphylococcus haemolyticus,* and *Escherichia coli* treated with leaf extracts from *Annona Crassiflora, Caryocar brasiliense,* and *Schinopsis brasiliensis* with (TA) or without (WT) tannins (1.58 mg/mL)

Bacteria Strains^a^	*A. crassiflora* Ethanolic-		*C. brasiliense* Ethanolic		*C. brasiliense* Aqueous		*S. brasiliensis* Ethanolic		*S. brasiliensis* Aqueous	
	TA	WT	TA	WT	TA	WT	TA	WT	TA	WT
S135	7.3 ± 0,67 Dd	0	8.1 ± 0,11 Ce	0	6.1 ± 0,08 Ed	0	9.4 ± 0,27 Bb	0	10.0 ± 0,18 Ac	0
S182	6.9 ± 0,38 Ce	0	6.5 ± 0,20 Dg	0	6.1 ± 0,11 Ed	0	9.3 ± 0,22 Bb	0	10.8 ± 0,33 Ac	0
S178	8.6 ± 0,47 Dc	0	8.4 ± 0,04 Dd	0	6.1 ± 0,11 Ed	0	9.1 ± 0,29 Bb	0	9.8 ± 0,11 Ac	0
ATCC 25923	7.0 ± 0,06 Ee	0	7.3 ± 0,18 Df	0	9.4 ± 0,4 Cc	0	10.2 ± 0,22 Ba	0	15.1 ± 0,18 Aa	0
E2	9.8 ± 0,31 Ba	0	8.9 ± 0,18 Dc	0	14.1 ± 0,18 Ab	0	6.2 ± 0,07 Ec	0	9.3 ± 0,22 Cc	0
E3	9.5 ± 0,76 Cb	0	9.4 ± 0,27 Db	0	14.8 ± 0,27 Aa	0	6.1 ± 0,15 c	0	12.4 ± 1,44 Bb	0
ATCC 25922	10.1 ± 0,53 Da	0	11.3 ± 0,2 Ca	0	14.5 ± 0,44 Aa	0	8.5 ± 0,29 Ec	0	11.9 ± 0,22 Bb	0

Lowercase letters in lines indicate significant difference between bacteria strains and uppercase letters in columns indicate significant difference between plant extracts as determined by Scoott-Knott’test with a 5% significance

^a^ *S. haemolyticus* (S135 and S182); *S. aureus* (S178 and ATCC 25923) and *E. coli* (E2,E3 and ATCC25922)

The AE from *S. brasiliensis* produced larger inhibition zones against *Staphylococcus* spp. strains than other extracts. However, considering the *E. coli* strains, the *C. brasiliense* AE produced the largest areas of inhibition among all the extracts (Table 4, *P* < 0.001).

The MICs observed for the EEs from *S. brasiliensis* and *C. brasiliense* were lower than *A. crassiflora* EE. Both the MIC and MBC of the *A. crassiflora* EE were 6.24 mg/mL for all bacterial strains (Table 5).

**Table 5:** Minimum inhibitory concentration (MIC) and minimum bacterial concentration (MBC) of leaf extracts from *Annona Crassiflora, Caryocar brasiliense,* and *Schinopsis brasiliensis* tested on *Escherichia coli* and *Staphylococcus* spp. from cattle

Bacteria strains^a^	*Annona crassiflora*		*Caryocar brasiliense*				*Schinopsis brasiliensis*			
	Ethanolic mg/mL		Ethanolic mg/mL		Aqueous mg/mL		Ethanolic mg/mL		Aqueous mg/mL	
	MIC	MBC	MIC	MBC	MIC	MBC	MIC	MBC	MIC	MBC
*Staphylococcus* spp.										
S135	6.24	6.24	0.27	> 40.0	0.71	0.71	0.17	0.34	0.42	0.42
S182	6.24	6.24	0.27	> 40.0	0.71	0.71	0.17	0.68	0.42	0.84
S178	6.24	6.24	0.27	> 40.0	0.71	> 40.0	0.17	0.34	0.42	0.42
ATCC 25923	6.24	6.24	0.27	> 40.0	0.71	0.71	0.17	0.68	0.42	0.84
*Escherichia coli*										
E2	6.24	6.24	0.27	> 40.0	0.71	> 40.0	0.17	0.34	0.10	0.42
E3	6.24	6.24	0.27	> 40.0	0.71	0.71	0.17	0.34	0.42	0.42
ATCC 25922	6.24	6.24	0.27	30.0	0.71	> 40.0	0.17	0.34	0.42	0.42

^a^*S. haemolyticus* (S135 and S182); *S. aureus* (S178 and ATCC 25923) and *E. coli* (E2, E3 and ATCC25922)

### Reversed-phase HPLC characterization of selected plant extracts

**Fig. 1:**
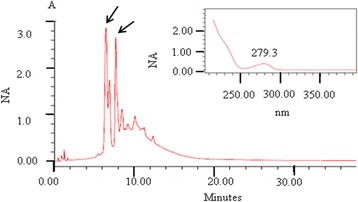
HPLC chromatographic profile, retention times (RT), and UV (279.3 nn) spectrum characteristics of flavonoids, in panels inside the image, in the ethanolic extract from *Annona crassiflora* (first RT = 6.484 min).

**Fig. 2:**
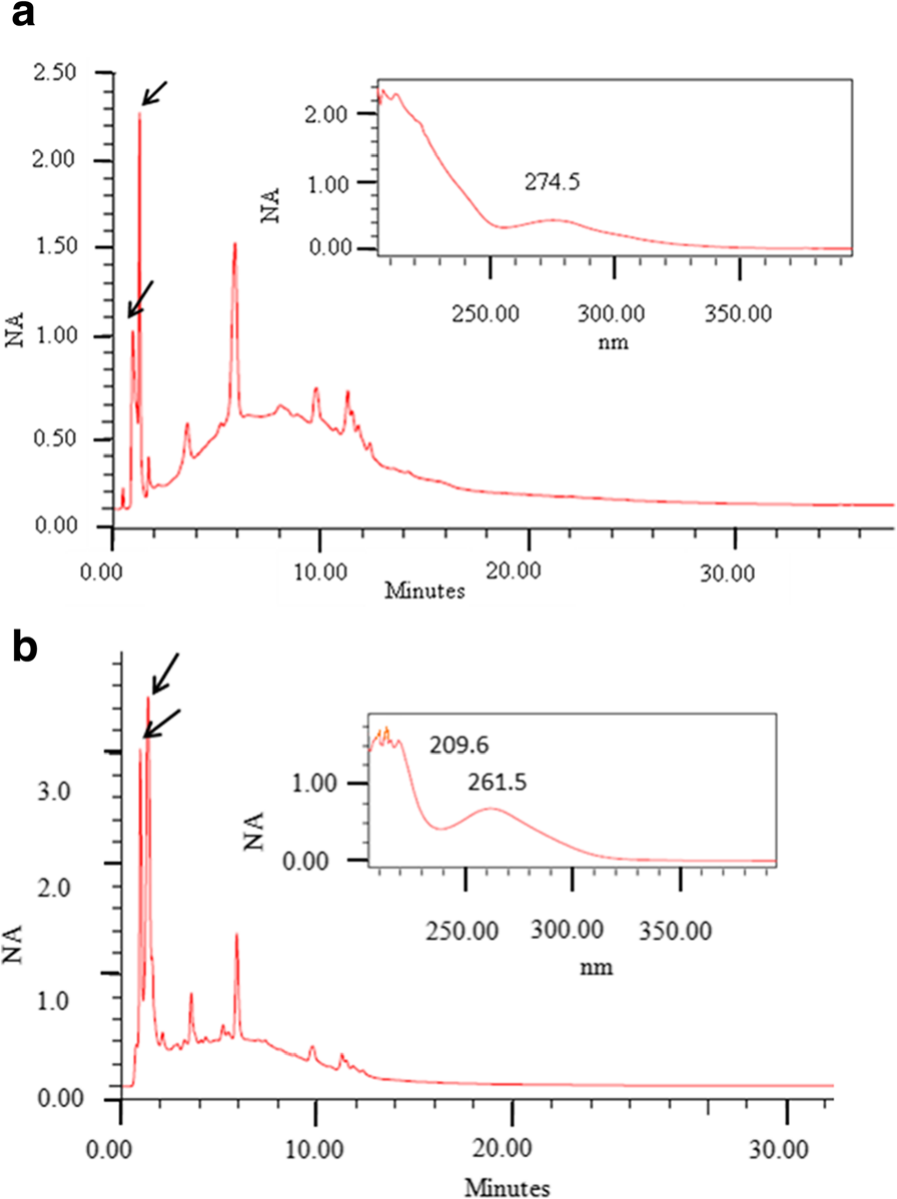
HPLC chromatographic profiles, retention times (RT), and UV spectrum characteristics of flavonoids in extracts: **a** *Caryocar brasiliense*, ethanolic (RT = 1.284 min and UV =274.5); **b** *C. brasiliense*, aqueous (RT = 1.378 min and UV=261.5)

**Fig. 3:**
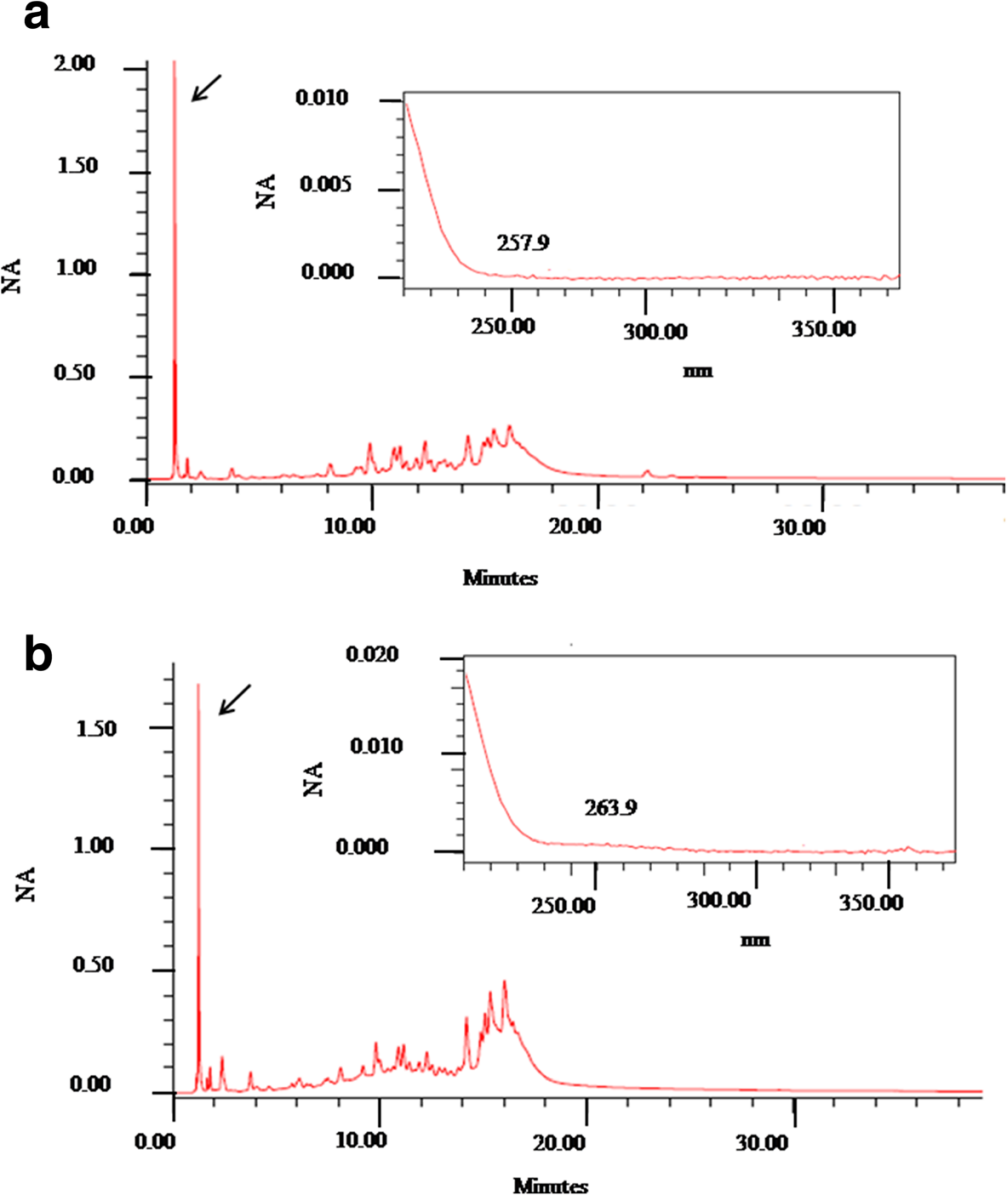
HPLC chromatographic profiles, retention times (RT), and UV spectrum characteristics of tannins in extracts: **a** *Schinopsis brasiliensis,* ethanolic (RT = 1.053 min and UV = 257.9)*;* **b** *Schinopsis brasiliensis,* aqueous (RT = 1.054 min and UV=263.9)

According to the UV spectra observed, the presence of flavonoids was detected in the region between 261 and 279.3 nm for the EE from *A. crassiflora* (Fig. 1), and EE and AE from *C. brasiliense* (Fig. 2). Tannins were detected in the EE and AE of *S. brasiliensis*, with absorbance at 257–263 nm for the respective retention times (Fig. 3).

## Discussion

In this study, six plants showed inhibitory effect against the three bacterial species evaluated, indicating the importance of bioprospecting studies on Cerrado vegetation. We observed intra- and inter-species differences in the bacterial susceptibility to plant extracts, which should be clarified in future investigations.

The differences in inhibition zones produced by the five selected extracts could be associated with their biochemical compositions, polarity, and solubility in Muller-Hinton agar. Additionally the interactions and the differences of extract constituents of the extracts could explain their spectra of action against Gram-positive or Gram-negative bacteria.

In agar diffusion test, the best inhibitory action against the Gram-negative *E. coli* strains was promoted by *C. brasiliense* AE which contained flavonoids. However, the AE from *S. brasiliensis,* containing tannins, produced larger inhibition zones in the Gram-positive *Staphylococcus* spp. strains than did other extracts. We suggested intra-specific variations in bacterial response to the extracts, considering the different effects of these extracts against human clinical strains (*E. coli* ATCC 25922 and *S. aureus* ATCC 25923) and respective bovine isolates. The different hosts could lead to the selection of external or internal variations in these bacteria, which could influence their susceptibly to these extracts. However, this would need to be elucidated in future research.

A specific mechanism of action against *Staphylococcus* spp. or *E. coli* could be explained by differences in cell wall constitutions of Gram-negative and Gram-positive bacteria [27]. Specific constituents in *C. brasiliense* extracts such as saponins and flavonoids could be more toxic to walls with greater lipid contents, as was observed in *E. coli* [28, 29].

We detected that a lower number of the effective extracts against the Gram-negative bacteria. In another antibacterial screening of plants from the Cerrado revealed that the stem bark extracts from *Qualea grandiflora* Mart (Vochysiaceae), *Virola surinamensis* (Rol.) Warb (Myristicaceae), and *Hancornia speciosa* Gome (Apocynaceae) significantly inhibited *S. aureus* but were not effective against *E. coli* [13].

The results obtained in this research corroborate with those of the study by Lima et al. [30] that evaluated the in vitro antimicrobial activity of EEs (1:1) from leaves, fruits, and seeds of *A. crassiflora* against *Staphylococcus* spp. The authors showed that these extracts significantly inhibited bacterial growth with a mean inhibition zone of 10 to 12 mm. Silva et al. [31] also reported inhibition zones of 10 to 14 mm when analyzing the effects of *A. crassiflora* leaf extracts against multi-resistant human *S. aureus.*

Considering antibacterial effects of *C brasiliensis*, unlike the leaf extracts in our study, bark extracts used at 500 mg/mL showed no inhibition when tested against the reference strains of *S. aureus* and *E. coli* [32]. These results indicate the leaves, which are more available, as sustainable source of the antibacterial metabolites of this plant.

Analyzing the MICs observed in this research, the EEs and AEs from *S. brasiliensis* and *C. brasiliense* were more effective against all bacterial strains than *A. crassiflora* extract. However MBC values were different among the bacterial strains, thus showing intra-specific variations. The EE of *C. brasileiense* showed bacteriostatic effect while other extracts were bactericides.

Although of few scientific studies describe antagonistic effects of plant extracts against Gram-negative bacteria, we detected that the extracts from *C. brasiliense* and *S. brailiensis* had lower MICs against *E. coli* strains than other studies. Paula-Junior et al. [28] reported that the hydroethanolic extract from *C. brasiliense* leaves exhibited MIC values of 4 mg/mL against *E. coli* and *S. aureus,* and Amaral et al. [33] described higher MICs for *C. brasiliense* EE against *E. coli* ATCC 25922 (MIC of 11.25 mg/mL) and *S. aureus* ATCC 6538 (22.5 mg/mL).

In other study, low MICs have also been reported for *S. brasiliensis*, *E. coli* ATCC 9723, and multi-resistant *S. aureus* isolates*,* with values varying from 0.025 to 0.100 mg/mL, depending on the fraction of extracts [14].

In this study, the EE of *A. crassiflora* showed MIC and MBC of 6.4 mg/mL for all bacteria evaluated, indicating this extract is safe and can ensure complete inhibition or death of these microorganisms. Silva et al. [31] evaluated the effects of leaf extract of this plant against oxacillin-resistant human *S. aureus* and ATCC 6538, and observed higher MICs (25 mg/mL) for both strains. Furthermore, the authors identified alkaloids and flavonoids as the active compounds.

Considering HPLC analysis, the EE of *A. crassiflora* and both extracts from *C. brasiliense* presented flavonoids while the tannins were main components detected for *S. brasiliensis* extracts. However, these analyses cannot reveal the concentration or chemical structure of these metabolites. The characterization of these extracts by other methods, such as gas chromatography after derivation, could reveal reveal the contents of their specific antibacterial components.

In this study, inhibitory effects were no observed after tannin removal from the selected extracts, indicating that this metabolite represented the main antibacterial agent in these extracts. Future studies should elucidate the mechanism of action of these extracts, and whether such effects can be linked to a group of substances promoting bacterial inhibition.

The antimicrobial role of these plant metabolites has not yet been clearly elucidated. However, there is a consensus of multiple mechanisms of action on bacterial cells. The tannins can interact with the cytoplasmic membrane, inhibiting its function and thereby compromising cellular integrity [11]. These compounds could also inhibit nucleic acid and enzymes synthesis, modify the cellular metabolism via membrane interaction, and complex with metal ions to decrease its availability for the microorganisms [34]. The antibacterial activity of flavonoids has been attributed to inhibition of DNA gyrase, inhibition of cytoplasmic membrane function, and energy metabolism. These compounds represent a novel source that can be utilized to develop pharmacologically acceptable antimicrobial agents [35].

Other studies have characterized the main metabolites of the plants selected in this research. Phytochemical tests of *C. brasiliense* identified condensed tannins, hydrolyzed tannins, flavonoids, terpenoids, and saponins, which could contribute synergistically with antibacterial effects [28, 29]. In the *A. crassiflora* extracts, alkaloids, acetogenins, flavonoids, and phenolic compounds have previously been detected [36].

We used the disk-diffusion assay for the antimicrobial screening of plant extracts which showed simplicity and low cost as reported by Balouiri et al. [37]. However, bactericidal and bacteriostatic effects were not distinguished and special attention should be given to the standardization of bacterial inocula and microbial procedures to reduce the variability of measures of the inhibition zones. The selected leaf extracts of native plants from the Cerrado showed inhibitory effects against three bacterial species related to mastitis or colibacillosis in cattle; both diseases that have caused significant economic loss in cattle production in several continents. The *E. coli* strains used were resistant to tetracycline and the S178 *S. aureus* isolate was multi-resistant. These results could be explained by bovine herds infected with *S. aureus* and *E. coli* being frequently treated with antimicrobials to control mastitis or colibacillosis [2, 3].

The selected extracts from native plants of the Cerrado could prove to be alternative agents for the control of colibacillosis, mastitis, and other diseases associated with these bacteria, after toxicity studies and in vivo tests are performed. The specific metabolites present in these extract could be essential to control resistant or multi-resistant bacterial strains.

## Conclusion

In this research, *Staphylococcus* spp. and *E. coli* were sensitive to leaf extracts of native plants from the Cerrado. Inter- and intra-specific bacterial variations were detected with regards to extract susceptibility. Notably, the *A. crassiflora* EE and *S. brasiliensis* extracts show potent bactericidal activity. After removal of the tannins, no antimicrobial effects were observed, indicating these metabolites are the main active antibacterial components.

## Abbreviation

AE: Aqueous extract
EE: Ethanolic extract
MBC: Minimum bactericidal concentrations
MIC: Minimum inhibitory concentrations
